# The Reduction of Uncertainties for Absolute Piston Gage Pressure Measurements in the Atmospheric Pressure Range

**DOI:** 10.6028/jres.094.033

**Published:** 1989

**Authors:** B. E. Welch, R. E. Edsinger, V. E. Bean, C. D. Ehrlich

**Affiliations:** National Institute of Standards and Technology, Gaithersburg, MD 20899

**Keywords:** manometer, piston gage, pressure, pressure measurement, pressure metrology, pressure standards

## Abstract

NIST pressure calibration services with nitrogen are now based on two transfer standard piston gages for which the effective areas have been determined by calibration with the manometer developed at NIST for gas thermometry. Root-sum-squared three sigma uncertainties for the areas for the two gages are 3.05 ppm and 4.18 ppm.

## 1. Introduction

There are presently only two technologies that can be developed into practical primary pressure standards in the atmospheric pressure range. They are the piston gage (pressure balance, dead weight tester) and the manometer.

The essential features of the piston gage include a vertical hollow cylinder which is closed at the top by a close-fitting piston and closed at the bottom with appropriate plumbing to admit the pressurizing fluid. The piston is loaded with known weights to counter-balance the effect of the pressure so that it floats at a specified reference level and is rotated to relieve friction. The pressure is calculated as the ratio of the force due to the weights to the effective cross-sectional area of the piston. The piston gage measures the difference between the pressure at the bottom and the top of the piston. When the top of the piston is at ambient atmospheric pressure, we speak of using the device in the gage mode. The absolute mode requires that the top of the piston be in a vacuum. The accuracy of a piston gage is limited by our ability to determine the effective area. For a primary standard piston gage, the area must be determined from dimensional metrology. The highest accuracy reported in the literature is on the order of 15 parts per million (ppm) [1] while repeatability of 1 ppm is common. The limitation is in the accuracy of the dimensional measurements and in the manufacture of truly straight and round cylinder bores and pistons.

The essential feature of a manometer is a vertical column of fluid, suitably contained and supported at the bottom by an applied pressure. The magnitude of the pressure is the product of the column height, the density of the fluid, and the acceleration due to gravity plus whatever pressure is applied to the top surface of the fluid column. Thus, the manometer is also a differential pressure measurement device and is used in the absolute mode when the space above the top surface of the field column is evacuated or in the gage mode when that space is at ambient atmospheric pressure. Limitations in accuracy are due to the uncertainties in the density of the fluid and in the column height measurements. In general, at atmospheric pressure, a state-of-the-art manometer will have a lower uncertainty than a state-of-the-art primary standard piston gage.

The piston gage has two advantages over the manometer: portability and ease of use. These two properties coupled with the stability of the piston gage make it an excellent transfer standard. In order to meet the demand for reduced uncertainties, NIST has used the manometer that was developed at NIST for gas thermometry [2] as a primary standard to calibrate the two piston gages that serve as the reference standards for NIST piston gage calibration service. Calibration services are now offered through these transfer standard piston gages.

Herein we report the results of the calibration of the piston gage calibration reference standards using the manometer in the absolute mode.

## 2. Apparatus

A complete description of the gas thermometer manometer (GTM) and a thorough evaluation of its uncertainties at the 99 percent confidence level are found in the literature [2,3,4]. Recently, Edsinger and Schooley, as a part of their new gas thermometry measurements up to 933 K, have reconsidered the uncertainty of the manometer and have determined that the value given in [2] of 2 ppm at the 99 percent confidence level is satisfactory [5].

The two piston gages are commercial units, identical in make and model. They were modified in two ways: in an effort to eliminate every part having even a remote possibility of being hard to clean and thus becoming a source of dirt, we replaced the entire mechanism for the top and bottom stops with parts made of Kel-F. Also the rotative mechanism was modified such that the cylinder and the piston with its weight stack could be rotated together up to the desired angular velocity, on the order of 60 revolutions per minute (rpm), and then the piston was floated and the cylinder was stopped allowing the piston to continue. Pressure measurements were then made with the cylinder stationary and the piston coasting. The Kel-F bottom stop was coated with a robust conductive aluminum film which was grounded to the cylinder to prevent electrostatic charges from accumulating on the surface when the rotating weight hanger rubs on the bottom stop. Without the conductive film, the resulting electrostatic forces can produce errors of several ppm at atmospheric pressure.

The temperature of the piston and cylinder assembly was monitored with an array of ten thermistors, connected in parallel, mounted on the stationary portion of the cylinder mount, and calibrated in situ.

**Figure 1:**
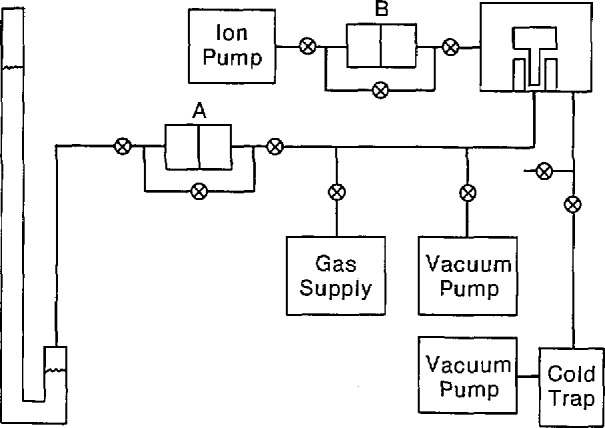
Schematic diagram of the pressure system connecting the manometer to the piston gage. The items marked A and B are calibrated differential pressure transducers. The circled X’s are valves.

Figure 1 is a schematic diagram of the experimental arrangement. Item A is a calibrated differential pressure transducer having a full range of 130 Pa and a sensitivity of 7×10^−2^ Pa. It was calibrated by using two piston gages, one supplying pressure to each pressure port of the transducer. The use of the transducer provides two advantages: it allows both instruments to work against a limited-volume pressure system which makes the apparatus easier to operate. The transducer was also used to read small differential pressures between the manometer and the piston gage which makes the establishment of a perfect equilibrium unnecessary. The zero was checked before each measurement by applying the identical pressure to both sides of the transducer via the bypass valve. Typically, the differential pressure for a given measurement was a fraction of a pascal.

Item B of figure 1 is another calibrated differential pressure transducer of the same make, model, range, and sensitivity as item A. It was used to measure the pressure in the bell jar surrounding the weights, which is evacuated for absolute mode operation.

The pistons and cylinders were examined for residual magnetism using a Hall-effect detector and were demagnetized as necessary.

## 3. Results

Both gages were repeatedly calibrated with the manometer at 27 and 95 kPa in the absolute mode using nitrogen; a total of 107 measurements for PG 28 and 149 measurements for PG 29. The effective areas for the gages were calculated from the expression

$$A=\frac{Mg}{\left(P-P_{b}-pgh+\epsilon \right)\left[1+\left(\propto_{p}+\propto_{c}\right)\left(T-T_{R}\right)\right]}$$ (1)

where

*M* is the total mass supported by the pressure including the weights and piston
*g* is the local acceleration due to gravity
*P* is the pressure at the lower mercury surface in the manometer
*P*_b_ is the pressure in the bell-jar surrounding the weights
*ρ* is the density of the nitrogen
*h* is the height between the reference level of the piston gage and the level of the lower mercury surface in the manometer
*ϵ* is the differential pressure measured by transducer A
∝_p_ is the linear thermal expansion coefficient for the piston
∝_c_ is the linear thermal expansion coefficient for the cylinder
*T* is the temperature of the operating piston gage
*T*_R_ is the reference temperature and is defined to be 23 °C.

**Figure 2:**
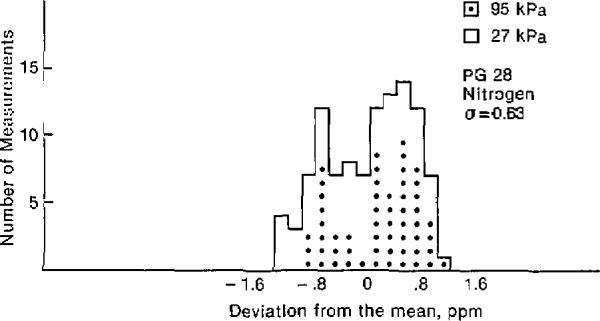
Histogram showing the deviation of the measured area from the mean for **PG** 28.

**Figure 3:**
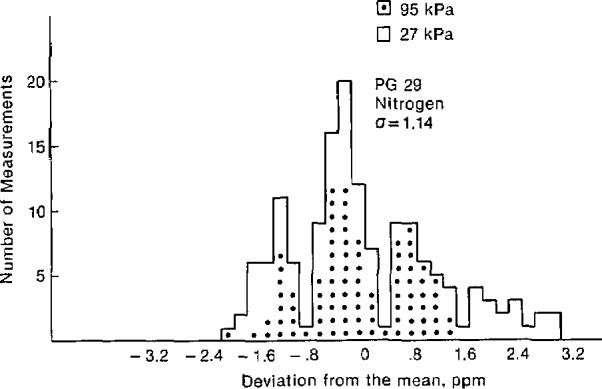
Histogram showing the deviation of the measured area from the mean for PG 29.

The effective area of each gage was found to be constant over this pressure range. Figures 2 and 3 are histograms showing the deviation from the average areas expressed in ppm. The measurements at 95 kPa are marked with a dot to distinguish them from the 27 kPa measurements. There is no apparent significant pressure dependence on the area of either gage. The areas and the tripled standard deviations are given in table 1.

The uncertainty in the effective area of each piston gage arising from the uncertainties in the various parameters of eq (1) can be expressed as [6]

$$\frac{dA}{A}={\left[\sum {\left(\frac{1}{A}\frac{\partial A}{\partial X_{i}}dX_{i}\right)}^{2}\right]}^{1/2}$$ (2)

where the *X_i_* are the parameters given in eq (1). These uncertainties are given in table 2. The tripled standard deviations about the means given for the areas of the two gages in table 1 are combined with the uncertainty given by eq (2) by the root-sum-square method; they are also listed in table 2.

The sources for the values listed under the heading “d*x_i_*” in table 2 are as follows:

The value of d*M* is the result of calibration of the piston gage weights using NIST standards and is discused elsewhere [7]. The value of d*g* is the result of on-site measurements. Reference 2 discusses the value of D*P.* The values of d*P*_b_ are based on calibration measurements. The value of d*ρ* is based on an uncertainty in temperature measurement of 0.5 K along the tube connecting the manometer to the piston gage [2]. On-site height measurements produced the value of d*h*. Separate thermal expansion coefficient measurements on the materials from which the piston and cylinders were made result in the value of d(∝_p_+ ∝_c_). The value for the uncertainty in the temperature measurements is based on calibration data of the thermistors.

The uncertainty in *P* includes the uncertainty in the head correction, *ρgh*, with the assumption that the gas is helium [2]. Since, in this case, nitrogen was used, we have explicitly included the uncertainty for the nitrogen head correction without removing the helium head correction uncertainty in the value of d*P*, a conservative approach.

The determination of effective areas of piston gages PG 28 and PG 29 as reported in this paper reflect state-of-the-art measurements and they provide an improved basis for NIST calibration services for the practical use of gas operated piston gages. It is recognized that reasonably frequent verification of the effective areas by the use of the GTM facility is important and necessary to establish a new body of control history to support improved levels of calibration service accuracy. The intrinsic stability of well constructed gas operated piston gages combined with the accuracy of the GTM provides a clear basis for significant improvement in the accuracy levels of practical pressure measurements.

## Figures and Tables

**Table 1:** Areas and tripled standard deviations of PG 28 and PG 29

	PG 28	PG29
Area, 10^−4^ m^2^	3.3582249	3.3572390
Triple standard deviation	1.89 ppm	3.42 ppm

**Table 2:** Uncertainties in area for gages PG 28 and PG 29 calculated at a pressure of 100 kPa based on three standard deviations

Parameter			Parameter Uncertainty	Differential		$\frac{1}{A}\frac{\partial A}{\partial X_{i}}dX_{i}$
*X_i_*	Units	Value	d*X_i_*	$\frac{1}{A}\frac{\partial A}{\partial X_{i}}$	Value	ppm
*M*	kg	3.4	1.1×10^−6^	1/*M*	3×10^−1^	0.33
*g*	m/s^2^	9.8	1.5×10^−6^	1/*g*	1×10^−1^	0.15
*P*	Pa	1×10^5^	2.0×10^−1^	1/*P*	1×10^−5^	2.0
*P*_b_	Pa	1.3	6×10^−2^	1/*P*	1×10^−5^	0.6
*ρ*	kg/m^3^	1.2	1.8×10^−3^	*gh*/*P*	5×10^−4^	0.9
*h*	m	5	2.0×10^−3^	*ρg*/*P*	1.2×10^−4^	0.24
ϵ	Pa	0.5	6×10^−2^	1/*P*	1×10^−5^	0.6
(∝_p_+∝_c_)	/°C	8.22×10^−6^	1.5×10^−8^	*T−T*_R_	1	0.015
*T−T*_R_	°C	*T*_R_ = 23	3×10^−2^	∝_p_+∝_c_	8.22×10^−6^	0.24
$\frac{dA}{A}$ arising from the parameters of eq (1)						2.40

				PG 28	PG 29	
	Uncertainty in eq (1)			2.40	2.40	
	Tripled standard deviation of area about the mean			1.89	3.42	
	Combined total uncertainty			3.05	4.18	
